# Facial landmark localization by curvature maps and profile analysis

**DOI:** 10.1186/1746-160X-10-54

**Published:** 2014-12-08

**Authors:** Carsten Lippold, Xiang Liu, Kim Wangdo, Burkhard Drerup, Kristina Schreiber, Christian Kirschneck, Tatjana Moiseenko, Gholamreza Danesh

**Affiliations:** Department of Orthodontics, University Medical Centre of Muenster, Waldeyerstraße 30, 48149 Münster, Germany; MOM, MAE, Nanyang Technological University, 50 Nanyang Avenue, Singapore, 639798 Singapore; Fac Motricidade Humana, CIPER, LBMF, SPERTLAB, Univ Tecn Lisboa, Estrada da Costa, P-1499-002 Lisbon, Portugal; Bundesfachschule f. Orthopaedietechnik (BUFA), Schliepstraße 6, 44135 Dortmund, Germany; Department of Orthodontics, Dental Clinic of Witten Herdecke, Alfred-Herrhausen-Straße 50, 58448 Witten, Germany; Department of Orthodontics, University Medical Center Regensburg, Franz-Josef-Strauß-Allee 11, 93053 Regensburg, Germany

**Keywords:** Orthodontics, Feasibility studies, Lasers, Models, Biological, Image interpretation, Reproducibility of results, Computer-assisted three-dimensional imaging, Lasers/diagnostic use

## Abstract

**Introduction:**

Three-dimensional landmarks of the face are important for orthodontic examination, harmony assessment and treatment planning. Currently, facial landmarks are often measured by orthodontists via direct observation and manual soft tissue image analysis. This study wants to evaluate and present an objective method for measuring selected facial landmarks based on an analysis of curvature maps and of sagittal profile obtained by a laser-scanning method.

**Methods:**

The faces of 15 people were scanned in 3D by means of the laser scanner FastSCAN™. It allowed the recording of a curvature map of the face in under a minute, which depicted the distribution of Gaussian and mean curvatures. The median-sagittal profile line of the face was localized in this map, and a mathematical analysis comprising its first and second derivatives was performed. Anatomical landmarks were identified and facial measurements performed. To assess validity the obtained data were compared with manual measurements by orthodontists by means of Lin’s concordance correlation CCC coefficient and reliability was determined by consecutive measurements.

**Results:**

Facial landmarks, such as the soft tissue glabella and nasal tip, could be easily and accurately identified and located. Lin’s CCC showed substantial agreement between digital and manual measurements for 4 of the 7 distances evaluated. Larger discrepancies were due to inadequate image quality and scanning errors. Reliability of consecutive measurements by the same operator was excellent.

**Conclusions:**

In our pilot study the three-dimensional laser-scanning method FastSCAN™ allowed a reliable and accurate identification of anatomical landmarks of the face. The obtained distances between certain landmarks, such as the intercanthal distance, were largely consistent with those from manual measurements. Due to its easy and rapid implementation, the method facilitates facial analysis and could be a clinically valid alternative to manual measurements, when remaining problems in scanning accuracy can be resolved.

## Introduction

The detection of three-dimensional landmarks by scanning surfaces is a well-established method in medical science. Anatomical surface landmarks of the face are visually or palpably detectable and can be used as reference points for clinical measurements. This is particularly important for orthodontists and maxillofacial surgeons, who are attempting to assess facial profile, harmony and balance. Clinical facial examination involving landmark localization within frontal and profile views of the face should be performed with less subjectivity [1]. Traditional soft tissue analyses record facial parameters separately for the three dimensions. However, when measuring soft tissue distances with a digital sliding caliper, no three-dimensional image is created. With surface laser scanners or parallel white light line projections, this shortcoming can be avoided [2]. An automated localization of landmarks would simplify an examination of the patient’s soft tissue profile considerably. Thus, this paper proposes and evaluates a new method for localizing important anatomical landmarks of the face and calculating facial parameters and distances required by orthodontists. This method aims to eliminate the subjective errors made by manual measurements and to simplify facial analysis by automatically obtaining landmarks and distances with a high level of accuracy and reproducibility.

## Methods

### Surface scanning and curvature maps

Measurements were performed with the FastSCAN™ laser-scanning system (Polhemus, Colchester, Vermont, USA) [3]. FastSCAN™ is a fast and convenient method to scan object surfaces providing 3D coordinates for surface points. The scanner took less than one minute for each surface scan of a human face. To generate a curvature map, each surface point was taken as a centre, and the curvature of that point calculated as follows: A neighbourhood of 6 mm × 6 mm was defined, in which all of the points were selected. The size of the neighbourhood was based on trial-and-error, and related to the point density and resolution requirement. A local coordinate system was established, taking the surface point as the origin and its normal direction as the z-axis. The 3D coordinates of the selected points were changed into the local coordinate system, which was prepared for a surface fitting. A second order six coefficient polynomial was fitted to this small patch using the least square method. The difference of z coordinates between the desired surface and selected points was minimized. From the six coefficients, the principal curvatures, mean curvature, Gaussian curvature [4] and Koenderink shape index [5] could be calculated.

**Figure 1:**
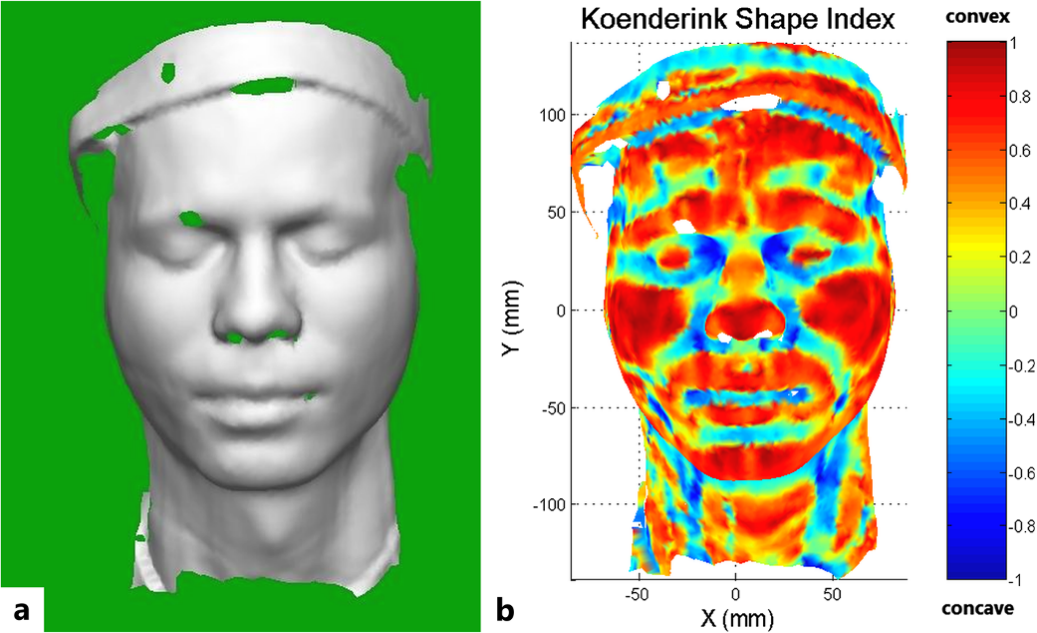
**Computer-generated images of the scanned facial curvature. a** FastSCAN™ curvature map of the facial surface. The hole within the eyebrow area is caused by laser absorption. **b** Koenderink shape index of the scanned face. Based on this index, several anatomical landmarks, such as the philtrum, the inner and outer corners of the eye and the corners of the mouth can be located.

The surface fitting and curvature calculation were performed for each surface point until the entire face was analysed. Within approximately 30 seconds, a curvature map of the face could be created (Figure 1a). The convex and concave areas of the face were differentiated within the map by means of different colouring, according to their Koenderink shape index (the indicator of whether the region is convex, concave or saddle-shaped).

The Koenderink shape index map of the face (Figure 1b) immediately showed several interesting regions, such as the philtrum, the inner and outer corners of the eye and the corners of the mouth. Although these structures cover a certain area, they can be defined as one single point each, according to the Koenderink shape index within a specific range by calculating the centre of gravity. For example, the localization of the philtrum was achieved by the following method: First, the rough position (0 < x < 10, -40 < y < -30) of the philtrum needed to be indicated manually by the researcher. The program then screened this region for surface points with a Koenderink shape index between -0.2 and -0.1. The centre of gravity of these selected points was then defined as the final position of the philtrum. The individual range for the different anatomical landmarks was determined by a trial-and-error method as well as by comparing the obtained results of the curvature analysis to a photographical image of the face. The range varied to a certain degree from subject to subject, but remained sufficiently stable for reliable landmark identification. The accuracy of the anatomical landmark localization has been shown previously [6, 7].

### Profile analysis and landmark identification

To assess the sagittal facial profile, a reproducible sagittal lateral profile line of the facial contour had to be determined. To this end, we first set the median centreline of the face through the philtrum point and the centre point of the connection line of the inner eye corners. Next all data points of the face within a distance of 0.5 mm to this starting reference line were selected. A plane was fitted to these selected points by means of the least square method. The intersection of this plane with the facial surface yielded the symmetry line of the face (the lateral profile line).

**Figure 2:**
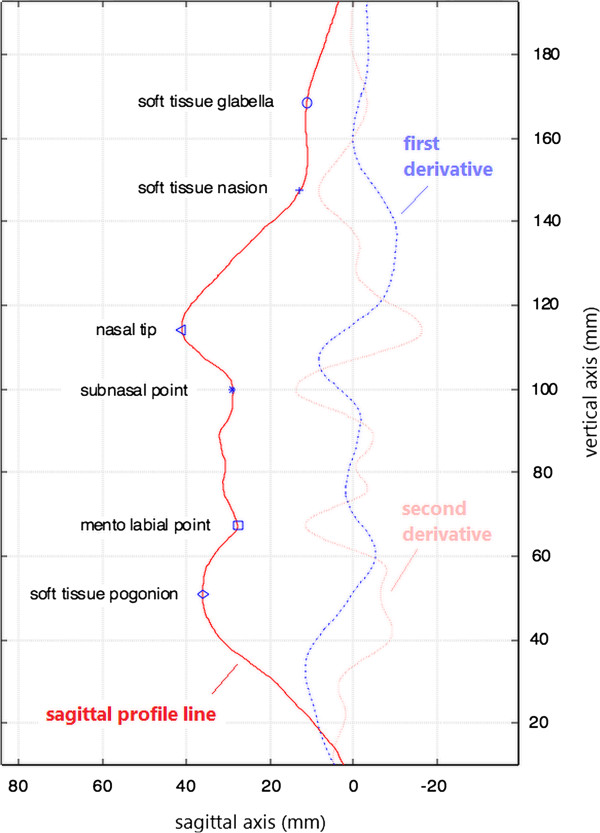
**Anatomical landmarks determined by the sagittal profile analysis.** The first and second derivatives are multiplied within the graph by factors of 10 and 100, respectively, to facilitate visual interpretation.

The obtained lateral profile line of the face was then converted into a mathematical function and its first and second derivatives were calculated and smoothed by moving polynomial fitting (Figure 2). The first derivative, which denotes the local changing rate of the sagittal profile curvature, varied from scan to scan, but the second derivative, denoting the direction of curvature of the sagittal profile (positive/negative), remained rather stable. By identifying the local maxima and minima of the second derivative of the facial sagittal profile curve, the anatomical landmarks could be accurately pinpointed and used for distance measurements of orthodontic interest, such as the distance nasion to subnasal point.

No definite local extremum of the second derivate could be identified for the soft tissue pogonion. However, a reliable way to determine its position, which has been used in conventional orthodontic facial analysis before, is to construct a tangent to the precisely identifiable nasal tip and the soft tissue chin. The tangent point obtained at the chin can be regarded as the soft tissue pogonion with sufficient clinical accuracy.

### Evaluation of validity and reliability

To assess the validity of the proposed laser-scanning method for clinical facial analysis, the faces of 15 orthodontic residents and employees of the dental clinic of Muenster were scanned (10 females and 5 males; ages 21.8 - 40.6 years, mean 28.2 years, SD 5.5 years), and their curvature maps were calculated. Within the Koenderink shape index maps, the previously described landmarks were identified. Seven important distances, which are commonly used by orthodontists to evaluate treatment options and outcome, were derived from these anatomical landmarks and compared with manual measurements by an experienced orthodontist:

-  ID – intercanthal distance
-  ML – mouth length
-  NW – nasal width
-  OE – distance between outer eye corners
-  NN – distance soft tissue nasion to subnasal point
-  NM – distance subnasal point to mentolabial point
-  NP – distance subnasal point to soft tissue pogonion

One of the 15 subject’s faces was scanned three times on three different days by the same researcher to evaluate the reliability of the anatomical landmark localization and profile analysis.

### Statistical analysis

The statistical analysis was performed with the online NIWA statistical calculator [8]. To assess the validity of the new system compared to conventional manual measurements, we used Lin’s concordance correlation coefficient CCC. This coefficient, first proposed by Lin [9] and used for the evaluation of equivalence between methods and concordance of continuous data, has considerable advantages over alternative methods (*t*-test, ICC, Pearson’s r etc.) [8], and is robust with more than 10 pairs of data [9]. Since no scale is available in the literature for the level of agreement, we used the values proposed by NIWA [8], with p_C_ > 0.99 denoting an almost perfect agreement, 0.95 < p_C_ ≤ 0.99 a substantial, 0.90 ≤ p_C_ ≤ 0.95 a moderate and p_C_ < 0.90 a poor level of agreement between the digital and manual measurements. The study was arranged according to the Helsinki criteria and authorised by the local Ethics Committee of the Medical Faculty, Wesphalian Wilhelms University, Münster (Germany).

## Results

The distances obtained from the curvature maps of the FastSCAN™ laser-scanning system are consistent with the manual measurements (Table 1). Most of the errors or differences to manual measurements were below 1 mm and only a few extended to 2 mm. According to Lin’s CCC there was a substantial agreement between manual and FastSCAN™ measurements for the ID, OE, NM and NP distances and a moderate agreement for the NW distance. Poor agreement was found for the distances ML and NN.

**Table 1:** **Facial distances of 15 patients, obtained from digital curvature map analysis (FASTScan™) or from manual measurements by orthodontists**

Patient	Method	Facial distances (mm)						
		ID	ML	NW	OE	NN	NM	NP
1	FASTScan™	24.65	48.37	36.88	94.15	52.23	37.64	52.45
	Manual	24.62	46.98	38.22	95.04	52.00	36.80	54.00
2	FASTScan™	25.48	50.61	32.77	102.23	45.52	37.37	53.52
	Manual	25.54	50.61	30.85	103.21	43.80	35.20	51.60
3	FASTScan™	25.43	54.04	37.55	99.69	48.81	39.45	54.41
	Manual	24.93	55.28	37.18	99.49	47.60	40.10	55.20
4	FASTScan™	29.45	49.88	33.98	108.10	50.28	45.71	63.73
	Manual	29.97	51.88	34.96	110.01	47.70	45.40	61.80
5	FASTScan™	24.05	51.59	29.78	92.85	53.68	41.90	60.52
	Manual	23.70	52.54	30.85	92.95	51.60	42.50	60.90
6	FASTScan™	24.35	50.87	36.05	95.74	49.23	38.10	54.09
	Manual	24.43	49.45	36.31	95.56	47.80	37.80	53.40
7	FASTScan™	24.99	52.96	35.88	102.49	55.10	39.41	57.74
	Manual	24.65	52.87	37.33	102.78	53.40	40.10	56.20
8	FASTScan™	26.61	54.55	32.96	98.39	50.40	38.56	60.73
	Manual	27.72	53.27	34.28	100.21	49.50	40.20	60.40
9	FASTScan™	29.52	54.03	35.57	101.94	46.14	47.81	61.73
	Manual	28.80	53.80	35.61	102.98	45.00	47.90	62.20
10	FASTScan™	27.34	39.42	34.99	111.01	51.25	45.74	64.65
	Manual	26.92	47.46	36.44	112.91	52.70	47.40	65.10
11	FASTScan™	31.05	57.29	32.42	103.27	56.37	40.79	60.95
	Manual	31.24	55.35	34.10	105.28	51.70	40.40	60.90
12	FASTScan™	28.48	53.37	29.79	97.21	47.44	42.61	63.64
	Manual	30.00	53.45	30.52	98.67	48.90	41.60	62.70
13	FASTScan™	22.05	51.44	31.51	96.35	44.34	37.25	53.39
	Manual	22.43	51.50	32.20	94.89	46.10	38.20	52.30
14	FASTScan™	23.23	50.20	31.21	101.68	57.00	40.04	58.31
	Manual	21.74	49.06	31.97	101.81	55.40	38.30	57.30
15	FASTScan™	21.85	48.18	30.82	89.68	47.69	44.60	57.15
	Manual	22.99	48.90	30.87	89.78	46.40	45.60	58.70
Lin’s CCC	p_C_	0.963	0.752	0.910	0.978	0.857	0.951	0.961

ID = intercanthal distance; ML = mouth length; NW = nasal width; OE = distance between outer eye corners; NN = distance soft tissue nasion to subnasal point; NM = distance subnasal point to mentolabial point; NP = distance subnasal point to soft tissue pogonion; CCC = Lin’s concordance correlation coefficient; p_C_ = value of CCC.

**Figure 3:**
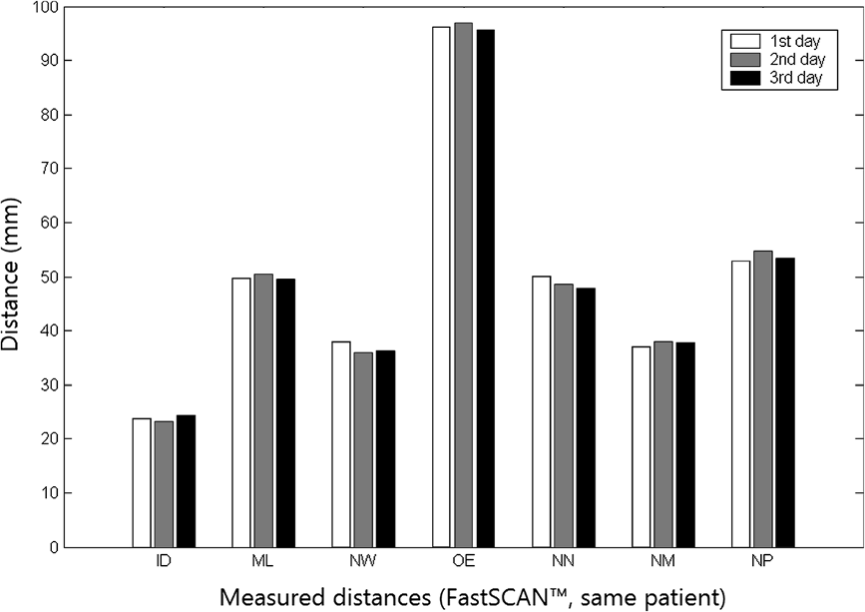
**Reliability of consecutive FastSCAN™ distance measurements.** Almost identical values were obtained from the three consecutive facial scans of the same patient for each of the seven distances evaluated.

The reliability of the FastSCAN™ measurements was excellent for all seven distances evaluated in this study. The three facial scans of a single patient on three consecutive days yielded almost identical values for all seven distances evaluated in this study (Figure 3). The reproducibility of locating the anatomical landmarks was demonstrated previously [6, 7].

## Discussion

The evaluation and analysis of facial soft tissues is an essential part of orthodontic diagnosis and treatment planning. Stoner [10] was one of the first to present an image-based analysis for soft tissue diagnosis. Since that time many manual analytic methods for soft tissue evaluation have been published, all with the purpose of obtaining quantitative information about facial soft tissues. These soft tissue analyses largely rely on two-dimensional photographic and radiographic images. Although these two-dimensional images have a long and proven history in orthodontics, they rely on two-dimensional lateral or frontal views. Thus the true curvature of the face in three dimensions is not considered. In recent years, as three-dimensional methods have become more accessible due to their reduced cost and greater applicability, the desire to implement 3-D images of the face as the preferred method for capturing and quantifying craniofacial morphology has become more popular.

Various techniques for the 3-D documentation of facial surfaces have been developed, such as laser scanning, holography and stereophotogrammetry. Laser scanning utilizes the detection of a reflected laser beam to reconstruct 3-D images. Thus, it is reliable and appropriate for the clinical assessment of facial morphometry in all three planes of space. Various studies have demonstrated that the use of laser scanning, which is a simple and minimal-invasive recording technique, provides a more accurate and precise analysis of craniofacial morphology, compared to the use of anthropometry, cephalometry, and imaging [11–14]. Optical laser scanning has been clinically used for the assessment of soft-tissue changes accompanying orthodontic treatment [15–18], as well as for the evaluation of nose and lip morphology in subjects with Down syndrome [19].

Soft tissue changes in various ways following orthodontic treatment have been reported, therefore a standardization of evaluation methods is necessary [20]. However, any evaluation of craniofacial morphometry is dependent on a realistic representation of facial details and the clinician’s ability to interpret the digital surface. Furthermore, for any evaluation, which is based on anatomical landmarks, the accuracy of reference point detection is vital for a future clinical usefulness and diagnostic relevance. If the identification and location of landmarks is imprecise, then the resulting assessment of an orthodontic outcome will be misinterpreted. Gwilliam et al. [21] observed that familiarity with software programs is likely to be an issue in the clinician’s ability to place landmarks. In their study on the reproducibility of soft tissue landmarks, they found that subjects, who were less familiar with the software, did not make use of additional functions, such as zoom, rotation and contrast enhancement. These researchers tended to view the facial image primarily from the frontal and profile view, which makes landmark detection more difficult. In their study, only the cheilion, labrale superius, and exocanthion points were found to be highly reproducible (SD 0.5 mm) for the intra-operator data. For the inter-operator data, none of the reference points were found to be highly reproducible for all three axes [21]. To avoid problems associated with variations due to observer subjectivity of identification and localization of landmarks, mathematically derived landmarks can be useful. Several attempts have been made to detect points of interest from the geometric information of 3D scans. However, none of these methods has yet gained widespread acceptance.

We performed this study to evaluate the accuracy of laser-scans for the measurement of clinically important landmarks and the distances between them by means of a novel automatic and objective method. Due to the pilot character of the study, which required total patient compliance, we chose to perform scans on participants, who had prior orthodontic knowledge and were properly instructed of the method to ensure minimal spontaneous movement of the head and face during the scanning process. This fact, however, should not have influenced the obtained results, since with sufficient instruction any patient should be able to minimize spontaneous movements during recording. We measured the intercanthal distance, mouth length, nasal width, outer eye corners distance, soft tissue nasion to subnasal point distance, subnasal point to mento labial point distance and the distance between subnasal point and soft tissue pogonion. These seven facial parameters included in the analysis describe the vertical and transverse relationships in facial shape analysis. For the detection of these landmarks and the distance measurements between them, we used three-dimensional local shape descriptors to extract points of interest that were subsequently identified and labelled as anatomical landmarks. The method presented in this study can be taken as a first step of an automatic and objective method to measure the distances between soft tissue landmarks. Data collection was performed using FastSCAN™, which is a handheld laser scanner with a magnetic tracking system to automatically register and convert single scans into one virtual 3D model in real time.

Validity was evaluated by determining the difference of each digital measurement from direct manual measurements. In general, measurements obtained from the scanned faces were very similar to manual measurements based on the physical face with only a few major discrepancies. In addition, the reliability of FastSCAN™ measurements was excellent in repeated measurements of the same patient/face on three consecutive days by the same operator.

One reason for the poor concordance of digital and manual measurements in some cases and the subsequent rather low CCC values for the ML and NN distances may be inadequate image quality. Acquiring high-quality three-dimensional facial images is vital in this method. During the digitization process, the subject is required to remain still, while the scanner acquires details of the subjects’ head. Due to the slowness of the scanning procedure, inadvertent head movements are likely, which may lead to distortions of the scanned faces. There are methods to minimize the effect of inadvertent movements, but the shortcoming of the movement influence still persists. In addition, patients are scanned with their eyes closed due to safety issues related to exposing the eyes to the laser, which may interfere with any landmarks placed around the eyes [21]. In addition, black or brown eyelashes and eyebrows absorb the laser beam accidentally, resulting in empty spaces in the area around the eyelashes and eyebrows. Filling the holes via interpolation might alleviate this problem. From time to time, the poor quality of the scanned image requires a new scan. The inability to capture the soft tissue surface texture has also been reported as a potential shortcoming because it makes identification of landmarks that are dependent on surface colour difficult [20]. The introduction of an automatic method for the localization of soft tissue landmarks may compensate for the last described disadvantage. We believe that with the rapid development of the laser scanning technology, a face with fantastic details will be provided and existing drawbacks will be easily resolved in the near future.

Importantly, although manual measurements served as the reference method in our study, this does not suggest that they are measured without errors. Apart from errors due to the photographic two-dimensional representation of a three-dimensional structure, also inter- and intra-operator errors do occur in manual measurements due to slight variations in the identification of soft tissue landmarks. In particular, the identification of landmarks that are dependent on the shape of an area rather than a particular point within it is subjective and prone to error [22]. For instance, the soft tissue pogonion is defined as the most prominent point on the soft tissue chin in the midsagittal plane. To locate this point on a gently curving slope with no corners is tricky and error-prone. Divergences in the measurement of the distance between the subnasal point and soft tissue pogonion may be dependent on the inaccurate localization of soft tissue pogonion on the physical face. Similar problems occur when other soft tissue landmarks have been localized. The curvature map helps to easily and objectively locate these landmarks within the scanned face.

The location of the inner eye corners and mouth corners can slightly affect the central symmetry line used as starting reference line. Therefore we used the points within 0.5 mm distance to this central symmetry line and the least square method to determine a sagittal profile line that is rather robust to these variations. The smoothing of the first and second derivatives by polynomial fitting does slightly shift the located anatomical landmarks, but to a degree (< 0.5 mm, if the profile is not overly smoothed) that we consider acceptable.

## Conclusions

This pilot study is a first step towards an automatic and objective localization method of the anatomical landmarks and pertaining distances in clinical facial analysis. Due to the objectivity of the proposed automated FastSCAN™ laser-scanning method inter-operator errors can be avoided. In addition, the compared to manual methods substantially accelerated data analysis generates clinically interpretable facial distances within only a few minutes. Thus future efforts should focus on further increasing accuracy and reducing time for scanning and data analysis, while extending clinical tests to a larger patient collective.
